# Extraction of an ectopic supernumerary tooth through nasal cavity with piezosurgery under local anesthesia: A case report

**DOI:** 10.1002/ccr3.9221

**Published:** 2024-07-25

**Authors:** Ailimaierdan Ainiwaer, Bumairemu Yiminjiang, Wang Ling

**Affiliations:** ^1^ Department of Oral Surgery Clinic, The First Affiliated Hospital of Xinjiang Medical University (Affiliated Stomatological Hospital) Research Institute of Stomatology of Xinjiang Uygur Autonomous Region Urumqi China

**Keywords:** ectopic supernumerary teeth, local anesthesia, nasal approach, nasal cavity, piezosurgery

## Abstract

**Key Clinical Message:**

The ectopic supernumerary teeth (ST) commonly occur in the oral cavity. Ectopic ST in the maxilla can be extracted not only through an intraoral approach but also through a nasal approach.

**Abstract:**

The ectopic supernumerary teeth (ST) commonly occur in the oral cavity. We are reporting a case of a 23‐year‐old female patient with one ectopic ST in the anterior midmaxillary region. We extracted the ectopic ST under local tissue anesthesia through the nasal cavity using piezosurgery and minimally invasive extraction tools. During the operation, the adjacent structures remained intact, and the patient discomfort. This case indicates that ectopic ST in the maxilla can be extracted not only through an intraoral approach but also through a nasal approach.

## 1 INTRODUCTION

Supernumerary teeth (ST) refer to extra teeth beyond the normal dental formula. ST are typically found in the maxillary region, and commonly located in the upper incisor area.^1^, ^2^, ^3^, ^4^, ^5^ However, the occurrence of ectopic ST is exceedingly rare, with only up to 1% of cases being reported.^6^ The etiology of ST is not yet clear, potential causes include atavism, the dichotomy of tooth germ, hyperactivity of dental lamina and genetic factors.^7^, ^8^, ^9^ ST often lead to impacted, delayed, and abnormal eruption, root resorption, or compression and bending of adjacent permanent teeth in children.^2^, ^3^, ^10^ Furthermore, odontogenic cysts may form, which can negatively impact children's facial esthetics, chewing function and also mental health.^11^ Most of ectopic ST are recommended to be extracted.^3^, ^4^, ^5^, ^12^, ^13^, ^14^

Hauer et al.^15^ used a modified maxillary vestibular approach with subperiosteal intranasal dissection for extracting an impacted ST and concluded that this approach can reduce postoperative complications. The advantage of the nasal approach is the lower incidence of postoperative complications and shorter surgical time. In addition, the surgical wound, bone removal, and risk of damage to the roots of upper incisors and the nasopalatine neurovascular bundle are minimized.^16^ In this study, we successfully extracted the ectopic ST through the nasal approach.

Studies have shown that piezosurgery^17^ can selectively perform cutting functions. When piezosurgery encounters vessels, nerves, or other soft tissues, piezosurgery stops working. Some studies have concluded that piezosurgery reduces surrounding tissue damage, promotes tissue repair and stimulates cells, facilitates tissue metabolism, and promotes bone regeneration. Thus, it can significantly reduce postoperative pain and swelling.^18^, ^19^, ^20^, ^21^

Most previous studies^15^, ^22^, ^23^ extracted the impacted maxillary anterior ST with general anesthesia, with only a small number of cases treated with local anesthesia.^6^ There some complications of general anesthesia^24^ including aspiration, bronchospasm, postoperative nausea and vomiting, sore throat, enamel fracture, avulsion and crown fracture. In this present study, the ST was extracted under local anesthesia with no complications, and the patient was very cooperative. In this case, we aim to study the advantages of using piezosurgery to extract ectopic teeth through a nasal approach under local anesthesia.

## 2 CASE HISTORY/EXAMINATION

**FIGURE 1:**
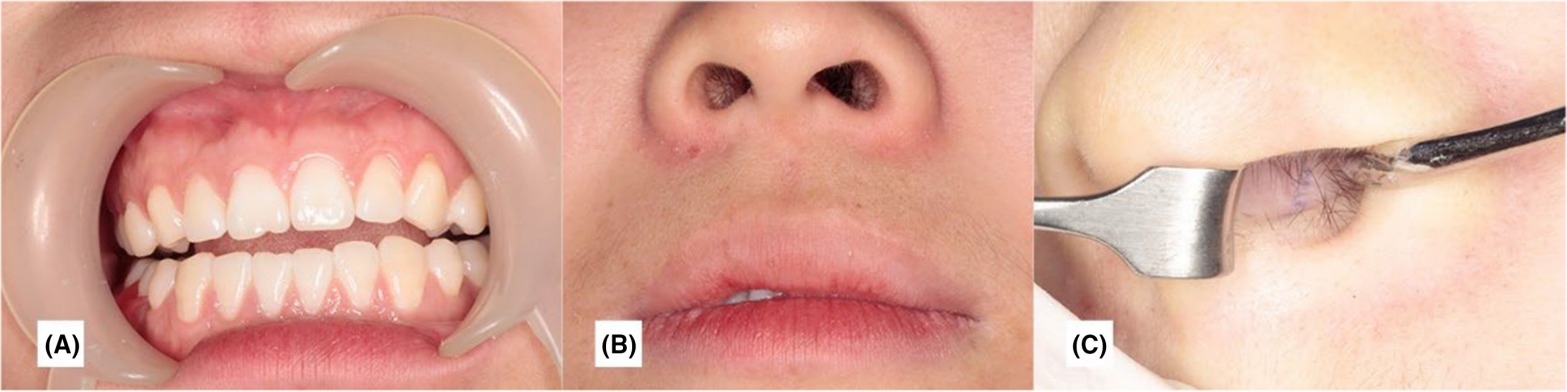
Preoperative photo of the patient.

A 23‐year‐old female patient presented to our department who was referred from the Department of Orthodontics to extract the ST. Upon examination, the ST were not palpable in the nasal cavity (Figure 1). The patient was asymptomatic and denied any pain, smell disturbances, postnasal drip, and previous history of maxillofacial trauma or surgery.

## 3 METHODS (INVESTIGATIONS AND TREATMENT)

**FIGURE 2:**
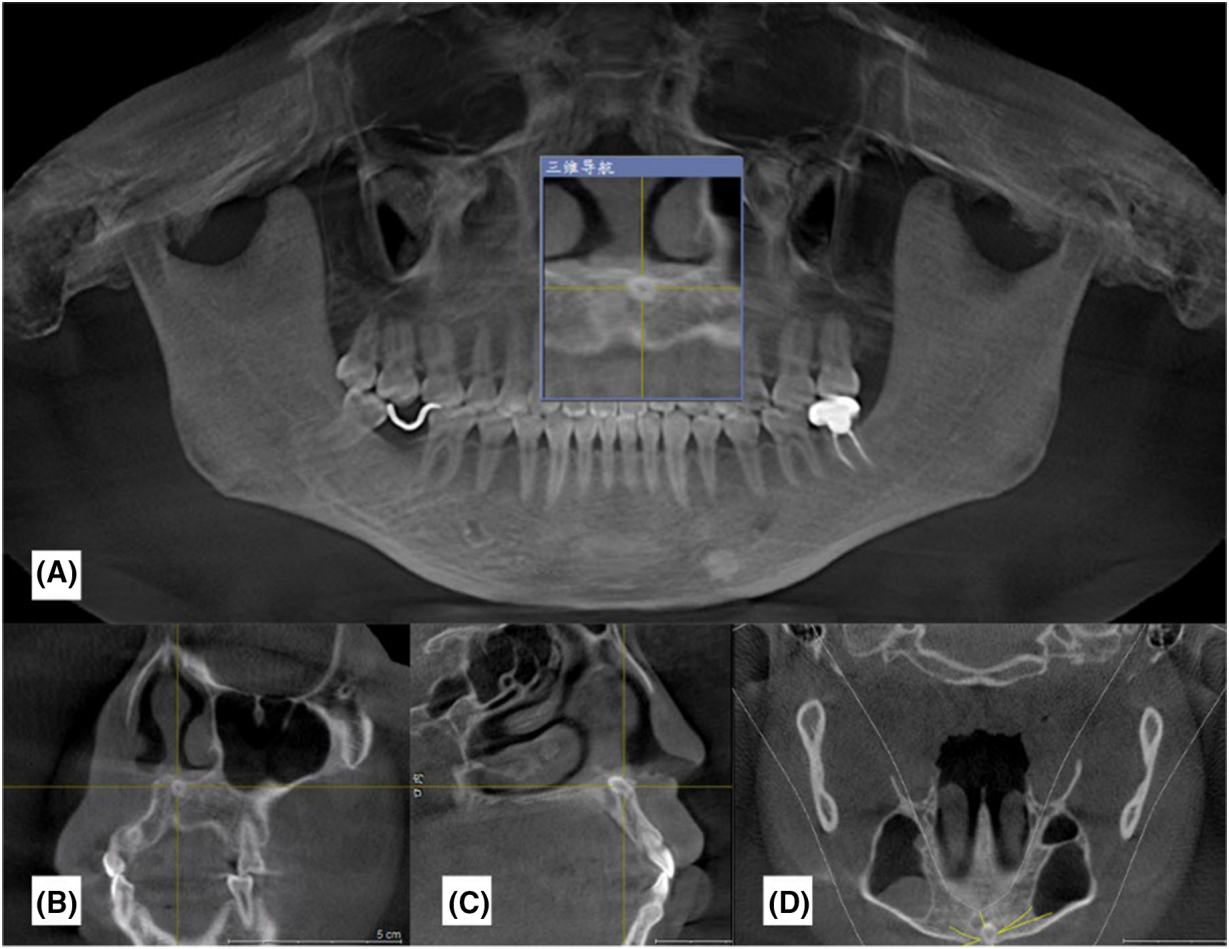
Preoperative CBCT images of the patient. (A)Preoperative pantomography; (B) cross section; (C) longitudinal section; (D) horizontal position.

Ancillary examination using cone beam computed tomography (CBCT) showed that the ST was visible at the tip of the left maxillary central incisor's root, which was extremely close to the incisal foramen and nasal floor (Figure 2).

**FIGURE 3:**
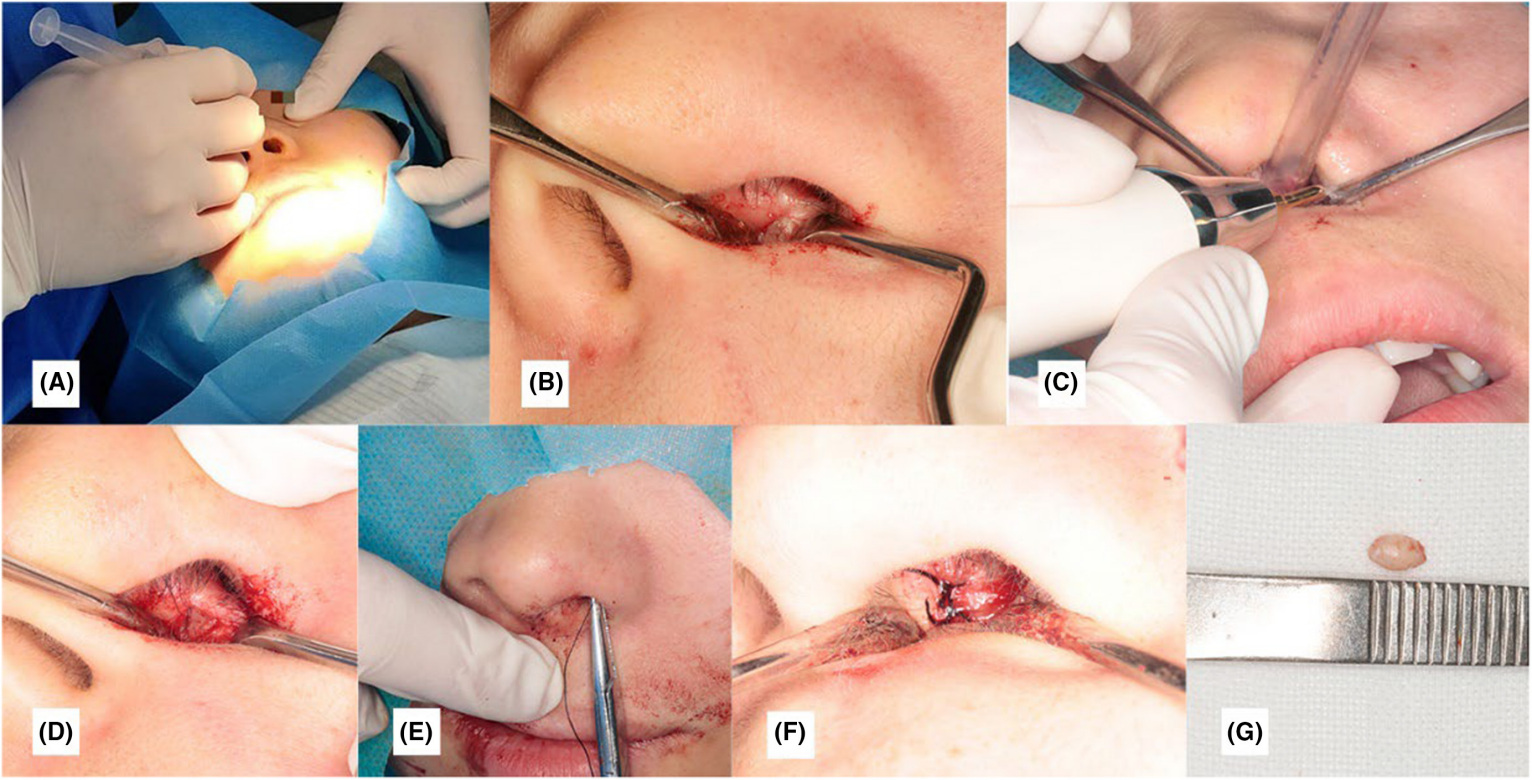
Operation process. (A) Infraorbital nerve block anesthesia was performed; (B) a surgical incision was made; (C, D) piezosurgery was used to remove bone after exposure of the operative area; (E, F) the wound was sutured after the ST extracted by a minimally invasive method; (G) the extracted ST.

Surgical Procedure: We performed an infraorbital nerve block anesthesia (Figure 3A). After the anesthesia took effect, we made a surgical incision (Figure 3B). After exposing the operative area, we used piezosurgery to remove bone (Figure 3C,D). We extracted the ST using a minimally invasive method and sutured the wound (Figure 3E,F).

## 4 CONCLUSION AND RESULTS (OUTCOME AND FOLLOW‐UP)

**FIGURE 4:**
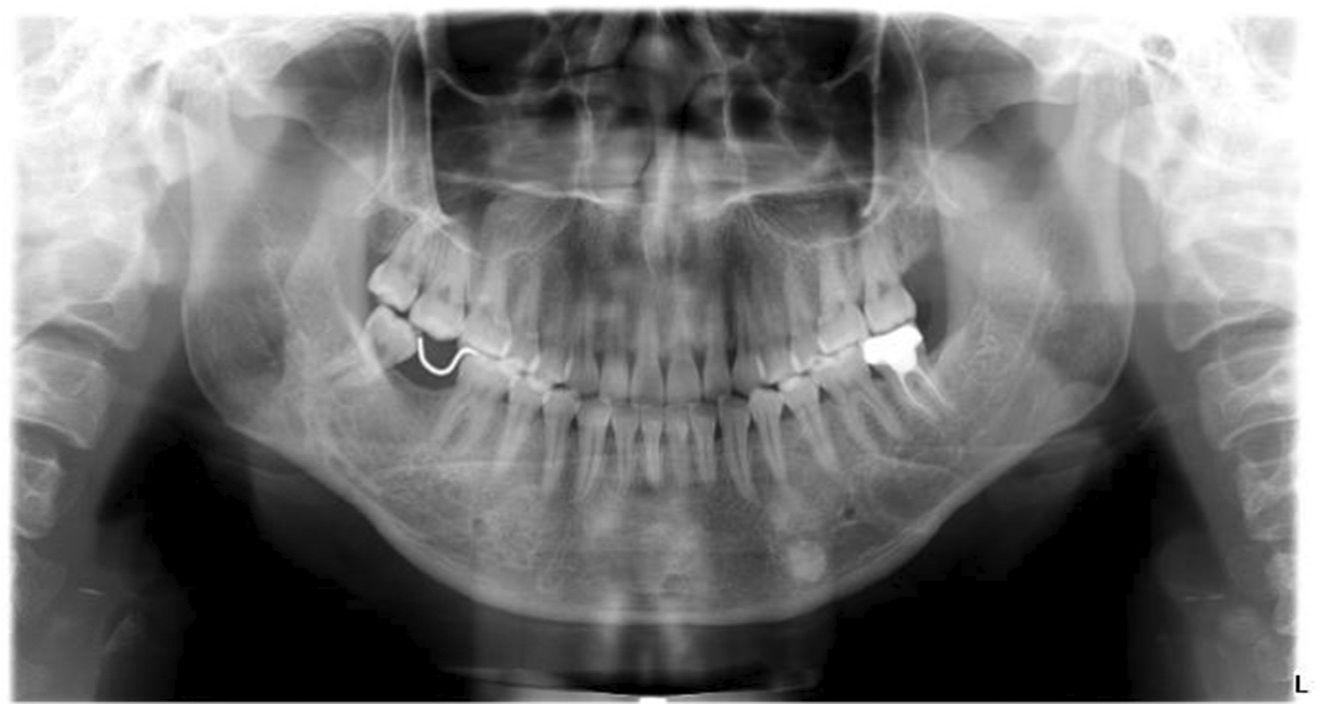
Postoperative pantomography.

After the extraction of ST (Figure 3G), we took a pantomography (Figure 4). The suture was removed 1 week after the operation.

## 5 DISCUSSION

During the extraction of an ectopic ST in the maxillary region, ST can be extracted from the nasal cavity in addition to the conventional intraoral surgical approach. According to a study,^16^ compared to conventional surgery, transnasal endoscopic approach had fewer postoperative complications and reduced operative time. In a study by M. Clementinile,^23^ a 9‐year‐old child had a ST extracted from the nasal cavity by means of an endoscopic approach, and it was concluded that the nasal cavity may provide another surgical approach. In our case, we extracted the embedded ectopic ST through the nasal approach, which reduced intraoperative bleeding and bone removal and alleviated postoperative complications.

Stbinger et al.^25^ found that the application of piezosurgery during the operation greatly reduces the amount of bleeding. Application of piezosurgery to the extraction of ST also can reduce postoperative pain, facial swelling, the amount of bone removal, and intraoperative bleeding, thereby relieving patient anxiety and fear.^21^ Similar conclusions were drawn in this case, as the patient experienced minimal intraoperative bleeding, less debridement and osteotomy, mild postoperative symptoms and good intraoperative cooperation.

In previous studies,^15^, ^16^, ^22^, ^23^ most of the ST was extracted using a transnasal endoscopic approach with general anesthesia. However, this approach may affect the nasal mucosa, such as causing injury to the nasal septum and resulting in massive nasal bleeding. General anesthesia may cause airway compromise or aspiration, and the postoperative reaction can be severe. We extracted the ST under local anesthesia through a nasal approach, which not only reduced the possibility of damage to adjacent teeth and nerves, but also reduced postoperative bleeding, pain, swelling, and other complications. Furthermore, it also shortened the patient's overall treatment period.

In the present case, extraction of an ectopic ST through the nasal cavity using piezosurgery under local anesthesia was performed successfully, with no other additional surgical procedures. During the operation, the adjacent structures remained intact, without any other complication and the patient was very cooperative.

## 6 CONCLUSION

Extracting ST through the nasal cavity has a low postoperative morbidity and a low risk of complications due to smaller surgical wounds, minimal exposure, less debridement and osteotomy, and reduced the risk of injury to upper incisor root and nasopalatine neuromuscular bundle compared to other techniques. This study demonstrated that the nasal cavity approach with piezosurgery is a useful approach for the exposure and removal of impacted ST, which reduces postoperative morbidity and provides a surgical approach for oral and maxillofacial surgeons.

## AUTHOR CONTRIBUTIONS

**Ailimaierdan Ainiwaer:** Operator of the surgery; conceptualization; writing‐review and editing. **Bumairemu Yiminjiang:** Writing – original draft; writing – review and editing. **Wang Ling:** Supervision.

## FUNDING INFORMATION

This research was funded by Youth Research Launch Project of the First Affiliated Hospital of Xinjiang Medical University (grant no. 2022YFY‐QKQN‐33).

## CONFLICT OF INTEREST STATEMENT

All authors declare no conflicts of interest.

## CONSENT

Written informed consent was obtained from the patient to publish this report in accordance with the journal's patient consent policy.

## Data Availability

Data sharing not applicable to this article as no datasets were generated or analyzed during the current study.
